# Socioeconomic factors and access to home dialysis and early kidney transplantation across Europe

**DOI:** 10.1007/s40620-025-02424-0

**Published:** 2025-09-19

**Authors:** Jan Dominik Kampmann, Vianda S. Stel, Leah Sejrup Christensen, Anneke Kramer, Patrik Finne

**Affiliations:** 1 University Hospital of Southern Denmark, Internal Medicine Research Unit, Sydvang 1, 6400 Sønderborg, Denmark; 2 Department of Regional Health, University of Southern Denmark, Odense, Denmark; 3 ERA Registry, Department of Medical Informatics, Amsterdam UMC – Location University of Amsterdam, Amsterdam, Netherlands; 4 Amsterdam Public Health Research Institute, Quality of Care, Amsterdam, Netherlands; 5 Nephrology Department, University of Helsinki and Helsinki University Hospital, Helsinki, Finland

**Keywords:** Home dialysis, Socioeconomic factors, Kidney transplantation, Peritoneal dialysis

The number of patients requiring kidney replacement therapy (KRT) is projected to reach at least 5.4 million worldwide by 2030 (S1). Kidney failure presents major challenges for patients and places a substantial financial burden on healthcare systems and societies [1] (S2). Home hemodialysis (HHD) and peritoneal dialysis (PD) offer greater autonomy and better quality of life compared to in-center hemodialysis (ICHD), often with lower economic costs [2] (S3, S4). Early kidney transplantation is generally superior in terms of mortality, quality of life, and healthcare expenditures [3]. Despite these benefits, adoption of home dialysis and kidney transplantation remains disproportionately low compared to ICHD and varies greatly across countries [1, 2] (S4). Barriers include lack of awareness, low health literacy, and socioeconomic factors [4, 5]. While disparities between countries are well recognized, it remains unclear whether national wealth, measured by gross domestic product (GDP) per capita or healthcare expenditure per capita, is associated with improved access to home dialysis or early transplantation. This study aims to estimate the association of GDP and healthcare expenditure with the adoption of home dialysis and early kidney transplantation in countries reporting to the European Renal Association (ERA) Registry.

We analyzed the proportion of home KRT, defined as the proportion of all patients on home dialysis, i.e. peritoneal dialysis and HHD, or having a kidney transplant, out of all patients on KRT at day 91 from the start of KRT. We selected the 91-day interval following KRT initiation, as patients who begin dialysis acutely often lack the opportunity to choose home-based modalities. Data for the year 2021 were retrieved from the ERA Registry for 32 countries that had sufficient data for the analyses [6]. Data on the GDP and healthcare expenditure were retrieved from the World Bank 2021 database (S5). Linear regression was used to assess the unadjusted associations between explanatory variables and the proportion of home KRT at day 91 from the start of KRT.

On day 91 from the start of KRT in 2021, the total proportion of home KRT ranged from 0 to 44% across European countries, with home dialysis ranging from 0 to 34% and kidney transplantation from 0 to 15% (Supplementary Table 1). GDP per capita ranged from 4,828 USD to 93,446 USD across countries. For each 10,000 USD increase in GDP per capita, the proportion of home KRT increased by 3.68 percentage points (95% CI 2.49–4.88) (Fig. 1). In the lowest GDP quartile (< 10,000 USD), the average proportion of home KRT was 5%, while it was 31% in the highest quartile (> 53,000 USD).

**Fig. 1:**
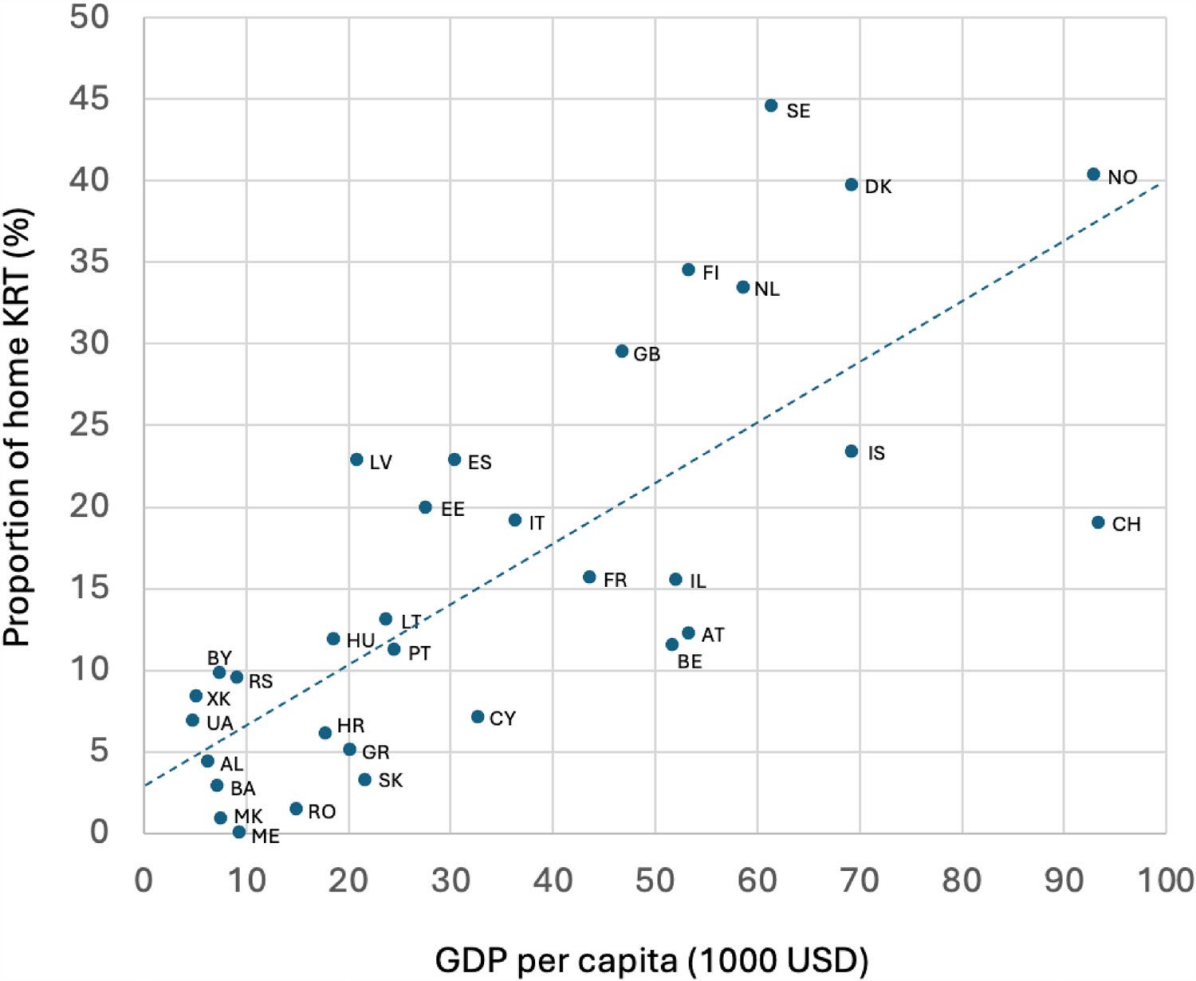
Scatter plot showing the correlation between the Gross Domestic Product (GDP) per capita and the proportion of home Kidney Replacement Therapy (KRT) at 91 days from the start of KRT. Country codes are explained in Supplement Table 1

Healthcare expenditure per capita ranged between 387 USD and 11,027 USD, strongly correlating with GDP per capita. For each 1,000 USD increase in healthcare expenditure per capita, the proportion of home KRT increased by 3.05 percentage points (95% CI 1.83–4.25). In the lowest expenditure quartile (< 1,000 USD), the average proportion of home KRT was 4%, compared to 30% in the highest quartile (> 6,000 USD). Our analysis did not reveal a statistically significant correlation between population density or population size and home KRT adoption (*r* = 0.03, *p* = 0.843 and *r* = 0.18, *p* = 0.154, respectively).

In a sensitivity analysis, the proportion of home dialysis increased by 2.63 percentage points (95% CI 1.63–3.63), and kidney transplantation by 1.05 percentage points (95% CI 0.64–1.47) for each 10,000 USD increase in GDP per capita. Population incidence of home KRT at 91 days ranged from 0 to 48 cases per million population (pmp). For each 10,000 USD increase in GDP per capita, the incidence of home KRT increased by 3.81 cases pmp (95% CI 2.44–5.17), with home dialysis increasing by 2.70 (95% CI 1.57–3.83) and kidney transplantation by 1.11 cases pmp (95% CI 0.62–1.59). On day 1 from the start of KRT, the proportion of home KRT varied from 0 to 43% across the countries. For each 10,000 USD increase in GDP per capita, the proportion of home KRT on day 1 increased by 3.77 percentage points (95% CI 2.60–4.93).

Our study showed that economically disadvantaged countries spending less on healthcare have considerably lower proportions of home dialysis and kidney transplantation at 91 days from the start of KRT. Poor socioeconomic status at the patient level has been recognized as a barrier to home dialysis and kidney transplantation [4, 5], but very few studies have addressed the association between country-level socioeconomic factors and utilization of home KRT.

A study using ERA Registry data found a negative correlation between healthcare expenditure as a percentage of GDP and the proportion of PD patients at day 91 from the start of KRT, after adjusting for GDP [7]. Our analysis found a positive link between absolute healthcare expenditure per capita and home KRT, echoing previous results. Both a European [8] and a global study [8] identified a positive correlation between GDP per capita and PD adoption, aligning with our observations.

How GDP may influence the proportion of home KRT and kidney transplantation at day 91 of KRT is uncertain. Late referral to nephrologists has surfaced as a potential explanation for the lower adoption of home dialysis and transplantation (S6, S7). The referral rate to nephrologists and specifically to home dialysis and transplantation appears to decrease even further in regions with lower income levels and countries with diminished GDP (S8, S9). A survey among European nephrologists suggested that barriers to home dialysis include staff shortage, lack of space and supplies, as well as less shared decision-making [9]. Some authors have suggested that the hemodialysis industry promotes the expansion of hemodialysis units and provides financial incentives, potentially contributing to a preference for ICHD over HHD and PD (S10). A recent study found that national guidelines supporting patient education in advanced kidney care, as a foundation for shared decision-making, were associated with higher prevalence of HHD and PD (S11). All of this may be linked to, or influenced by, national GDP. Although all countries with low GDP had a low home KRT adoption rate, high GDP does not always mean high adoption of home KRT, as evidenced by Switzerland’s comparatively low uptake despite its strong economy. This underscores that GDP is not an isolated determinant, but rather one of multiple interrelated factors, and that considerable variation exists among countries. Kidney transplantation emerges as the optimal treatment modality for most patients, offering superior outcomes in terms of morbidity, mortality, and quality of life compared to dialysis (S12). Financial barriers to kidney transplantation remain substantial both for individual patients and at a country level [8] (S13).

The strength of our study is that it is based on highly valid data from the World Bank database and the ERA Registry. However, we lacked data on factors that might explain the association between GDP and home KRT, such as details on healthcare policies regarding KRT on the national level. Furthermore, despite extensive checks at both the national/regional registries and the ERA Registry, we cannot fully exclude the possibility of treatment misclassification.

In conclusion, our findings indicate that the use of early home dialysis and kidney transplantation is low, especially in countries with lower GDP and healthcare expenditure. These observations underscore the importance for individual country-specific strategies in KRT provision grounded in a thorough understanding of each country's unique socioeconomic structure. Further research is needed to understand why countries with lower GDP and lower healthcare expenditure experience lower adoption rates for home dialysis and transplantation.

## Supplementary Information

Below is the link to the electronic supplementary material.

Supplementary file1 (DOCX 321 KB)

## Data Availability

This study utilizes only publicly available data, and as such, no ethical approval was required. All data sources are accessible to the public, ensuring transparency and reproducibility in the analysis.
