# Supplementary material for: Socioeconomic factors and access to home dialysis and early kidney transplantation across Europe
Source: J Nephrol. 2025 Sep 19;38(8):2469–71. doi: 10.1007/s40620-025-02424-0 (PMC12630190; doi:10.1007/s40620-025-02424-0)
Supplement: Supplementary file 1 — Supplementary file1 (DOCX 321 KB) [file 40620_2025_2424_MOESM1_ESM.docx]

**Supplement Table 1. Proportion of home KRT, home dialysis and kidney transplantation at 91 days from start of KRT and socio-economic determinants in 32 countries reporting to the ERA Registry in 2021.**

**
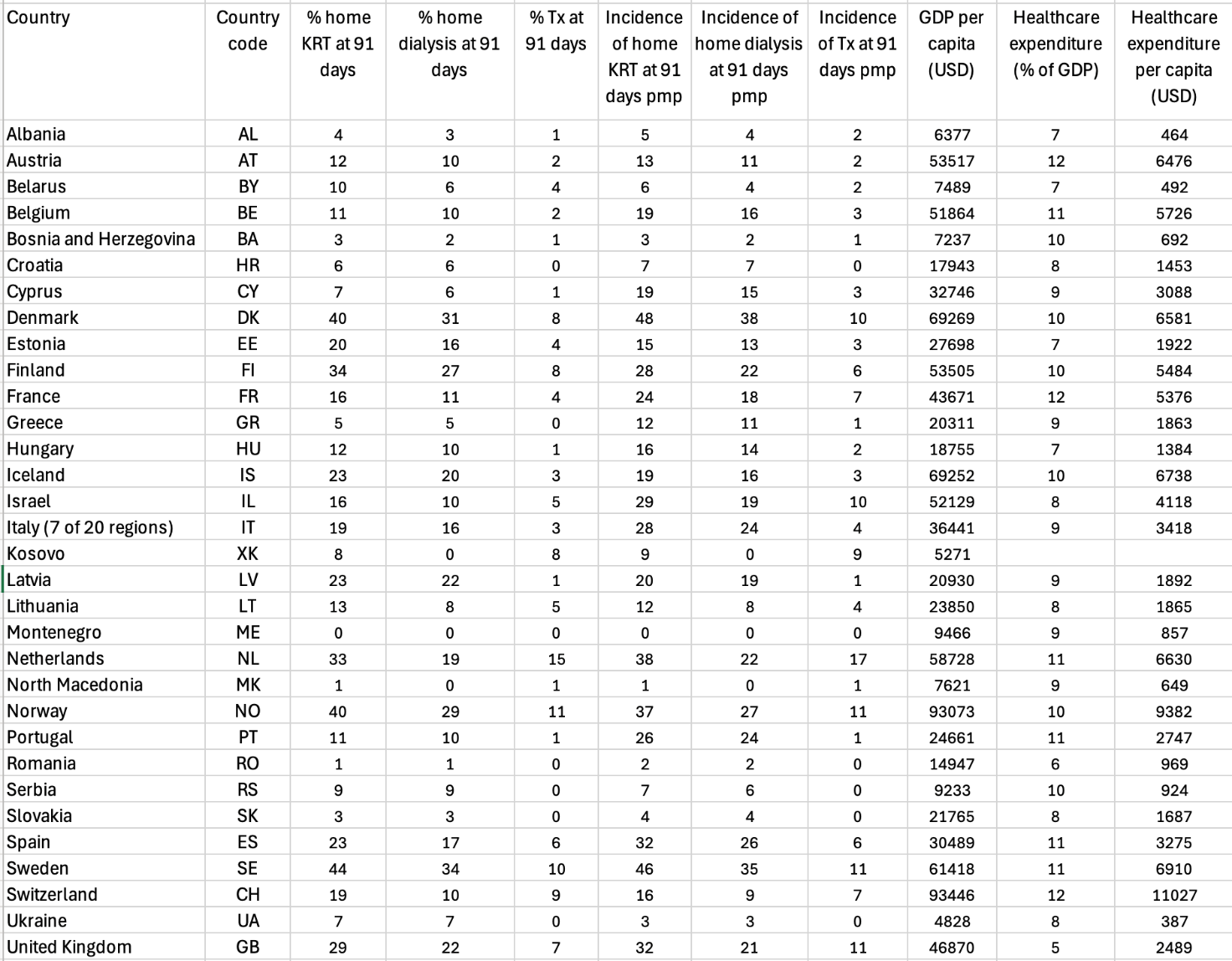
**

Supplement Table 1. Proportion of home KRT, home dialysis and kidney transplantation at 91 days from start of KRT and socio-economic determinants in 32 countries reporting to the ERA Registry in 2021.

Supplement references:

S1 Liyanage T, Ninomiya T, Jha V, Neal B, Patrice HM, Okpechi I, et al. Worldwide access to treatment for end-stage kidney disease: a systematic review. The Lancet. 2015;385(9981):1975-82.

S2 Alsing JFL, Hayes Bauer E, Brandt F, Kampmann JD. Expectations and Experiences of Patients Recently Initiated to Centre-Based Dialysis Treatment. Healthcare (Basel). 2022;10(5).

S3 Zhang Y, Gerdtham UG, Rydell H, Lundgren T, Jarl J. Healthcare costs after kidney transplantation compared to dialysis based on propensity score methods and real world longitudinal register data from Sweden. Sci Rep. 2023;13(1):10730.

S4 McFarlane PA, Pierratos A, Redelmeier DA. Cost savings of home nocturnal versus conventional in-center hemodialysis. Kidney Int. 2002;62(6):2216-22.

S5 https://data.worldbank.org/

S6 Kampmann JD, Heaf JG, Mogensen CB, Mickley H, Wolff DL, Brandt F. Referral rate of chronic kidney disease patients to a nephrologist in the Region of Southern Denmark: results from KidDiCo. Clin Kidney J. 2022;15(11):2116-23.

S7 Heaf J, Heiro M, Petersons A, Vernere B, Povlsen JV, Sørensen AB, et al. Choice of dialysis modality among patients initiating dialysis: results of the Peridialysis study. Clin Kidney J. 2021;14(9):2064-74.

S8 van Doorslaer E, Masseria C, Koolman X. Inequalities in access to medical care by income in developed countries. Cmaj. 2006;174(2):177-83.

S9 Thorsness R, Wang V, Patzer RE, Drewry K, Mor V, Rahman M, et al. Association of Social Risk Factors With Home Dialysis and Kidney Transplant Rates in Dialysis Facilities. JAMA. 2021;326(22):2323-5.

S10 Brown EA, Brivio GB, Van Biesen W. Towards a better uptake of home dialysis in Europe: understanding the present and looking to the future. Clin Kidney J. 2024 Jun 5;17(Suppl 1):i3-i12

S11 Lundström UH, Meeus G, Aronsen T, Clause AL, Finderup J, Finne PJ, Kampmann JD, Lange J, McCarthy K, Nohra R, Stompòr T, Wood E, Lichodziejewska-Niemierko M, Jacobson SH. Increasing the adoption of home dialysis through improved advanced kidney care patient education: a call for action. Clin Kidney J. 2025 Mar 27;18(4):sfaf087.

S12 Tonelli M, Wiebe N, Knoll G, Bello A, Browne S, Jadhav D, et al. Systematic Review: Kidney Transplantation Compared With Dialysis in Clinically Relevant Outcomes. American Journal of Transplantation. 2011;11(10):2093-109.

S13 Thorsness R, Wang V, Patzer RE, Drewry K, Mor V, Rahman M, et al. Association of Social Risk Factors With Home Dialysis and Kidney Transplant Rates in Dialysis Facilities. JAMA. 2021;326(22):2323-5.
